# Sirtuins mediate the reduction of age-related oxidative damage in the cochlea under a cocoa-rich diet

**DOI:** 10.1007/s11357-025-01847-8

**Published:** 2025-08-20

**Authors:** Rosalía Fátima Heredia-Molina, Juan Ignacio Riestra-Ayora, Israel John Thuissard Vasallo, Ricardo Sanz-Fernández, Carolina Sánchez-Rodríguez

**Affiliations:** 1 Department Clinical Analysis, Hospital Universitario de Getafe, Carretera de Toledo, Km 12.500, 28905 Getafe, Madrid, Spain; 2 Otolaryngology Department, Hospital Universitario de Getafe, Carretera de Toledo, Km 12.500, 28905 Getafe, Madrid, Spain; 3 Faculty of Medicine, Health and Sports, Department of Medicine, Universidad Europea de Madrid, Calle del Tajo S/N, 28670 Villaviciosa de Odón, Madrid, Spain

**Keywords:** Age-related hearing loss, Cocoa polyphenol, Oxidative stress, Sirtuins, Sex

## Abstract

Age-related hearing loss (ARHL) is a common condition among older adults. It has long been established that ARHL is more prevalent, is more severe, and tends to occur earlier in men than in women. A key factor contributing to ARHL is oxidative stress, which harms the inner ear cells. Sirtuins are a family of proteins that play critical roles in cellular regulation, particularly in response to stress and aging, and have demonstrated anti-aging effects. Cocoa flavonoids, due to their antioxidant properties, have been reported to provide numerous health benefits. The present study aimed to evaluate the efficacy of cocoa in preventing oxidative damage in the cochlea of aging mice mediated by Sirtuins, as well as to determine possible differences between sexes. Female and male C57Bl/6 J mice were randomly assigned to two study groups: standard diet and cocoa-supplemented diet. At the ages of 6, 14, and 22 months, the animals were sacrificed, and cochleae were harvested to measure the expression of Sirtuins-1, -3, forkhead box O3 (FOXO3), p53, antioxidant enzymes, oxidative DNA damage, and levels of reactive oxidative species (ROS). Our results indicated that cocoa attenuated oxidative DNA damage, reduced ROS levels, and induced the expression of SIRT1, -3, FOXO3, and p53 in the cochlea of ​​aged mice. Furthermore, the activity of antioxidant enzymes in aged mice was restored with the cocoa diet. Sex differences were observed in several of these variables. These results indicate that cocoa protects against oxidative damage through Sirtuins. In conclusion, cocoa may have therapeutic potential against ARHL.

**Graphical Abstract:**

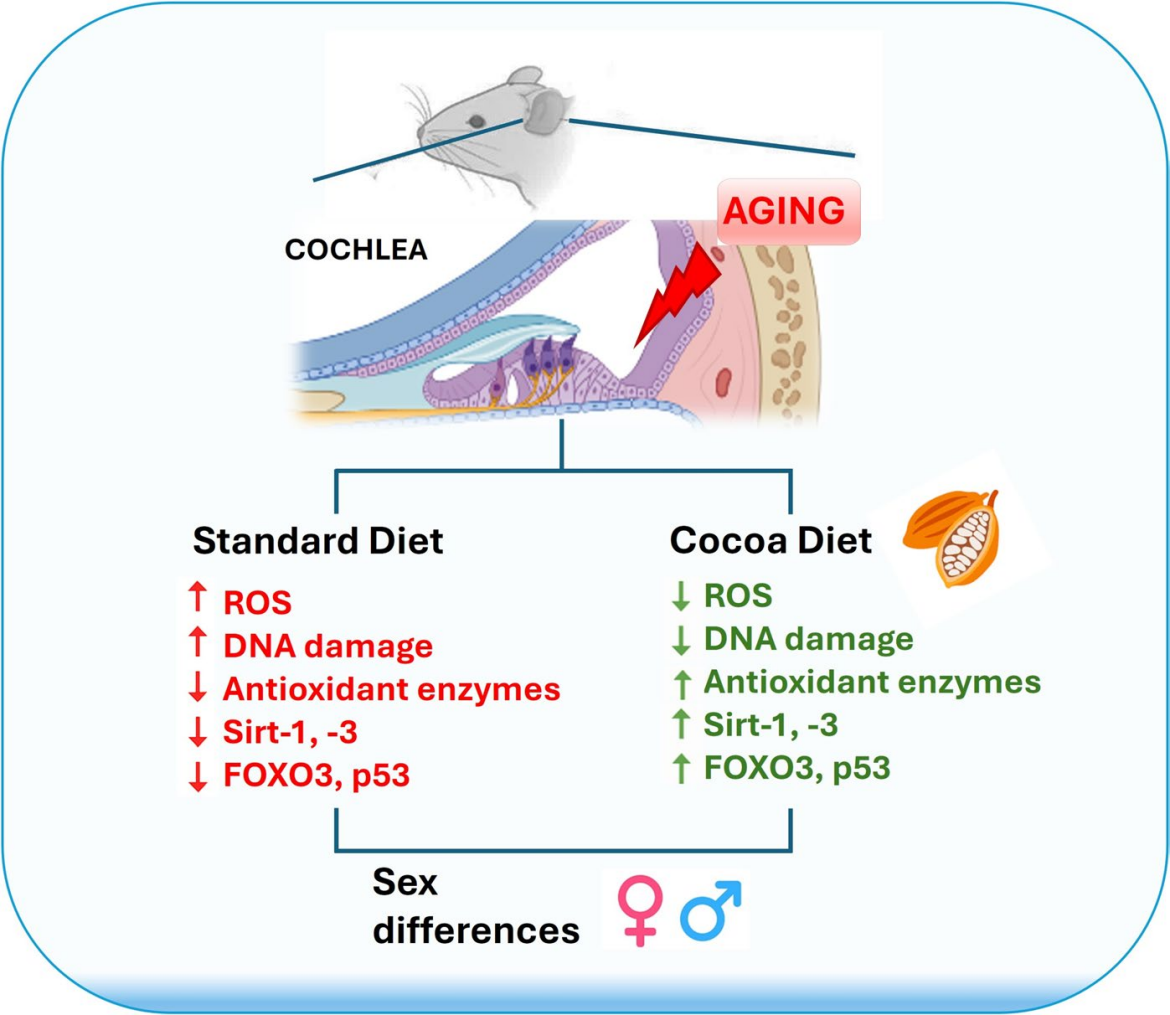

**Supplementary Information:**

The online version contains supplementary material available at 10.1007/s11357-025-01847-8.

## Introduction

Age‑related hearing loss (ARHL) is characterized by a gradual loss of hearing sensitivity, particularly at higher frequencies associated with age, no effective treatment available to date. ARHL, approximately, has a prevalence of 40% in the population over 65 years of age [1], reaching up to 80% in those over 80 years of age [2]. When the data is classified by sex, the prevalence of disabling hearing loss is higher in men in comparison to women, with global rates of 56% for men and 44% for women [3]. Recent findings from the Global Burden of Disease categorize hearing loss as the fourth leading cause of years lived with a disability, further underscoring its significance as an escalating economic burden [4]. Despite being frequently referred to as an invisible disability, hearing loss profoundly impacts the quality of life. Therefore, this will greatly increase the socioeconomic burden and research on new therapies for ARHL is urgently needed.

The accumulation of the effects of environmental factors (trauma, ototoxic agents, noise, immune system, vascular lesions, metabolic changes, or diet) and intrinsic factors (mitochondrial dysfunction, genetics factors, sex or oxidative damage) causes the development of ARHL [5, 6]. It has been widely acknowledged that aging is a process of cumulative oxidative damage [7].

Oxidative stress occurs when there is an imbalance between the production of reactive oxygen species (ROS) and the body’s ability to detoxify these reactive intermediates or repair the resulting damage using scavengers and antioxidant enzymes [8]. ROS damage various cellular components, including DNA, proteins, and lipids inducing cell dysfunction and death. Oxidative stress is a significant contributor to ARHL, as it accelerates the degeneration of cochlear hair cells, spiral ganglion neurons, and other structures essential for hearing [9].

Sirtuins family are (NAD +)-dependent class III histone deacetylases. The family is composed of seven members (SIRT1, SIRT2, SIRT3, SIRT4, SIRT5, SIRT6, and SIRT7) in mammals. Sirtuins are related to many pathophysiological conditions, such as oxidative stress response, apoptosis, cancer, aging, metabolism, proliferation, inflammation, and genome stabilization [10]. SIRT1 regulates various cellular processes, including aging, inflammation, and stress [11]. It has been shown to protect against oxidative stress by deacetylating and activating transcription factors like FOXO3a and p53, which are involved in antioxidant defense and DNA repair. SIRT1 also influences mitochondrial biogenesis and function, which are crucial for cellular energy metabolism and reducing oxidative damage [11].

SIRT3 is primarily located in the mitochondria and is crucial for maintaining mitochondrial integrity and function [12]. SIRT3 activates several mitochondrial enzymes involved in antioxidant defenses, such as superoxide dismutase 2 (SOD2) and isocitrate dehydrogenase 2 (IDH2), which helps to reduce the production of ROS and improve cellular resistance to oxidative stress [12].

Different studies have shown the protective effects of SIRT1 and SIRT3 in ARHL [13–19]. Therefore, modulating the activity of SIRT1 and SIRT3 presents a potential therapeutic approach for preventing or mitigating ARHL. Interventions that increase SIRT1 and SIRT3 activity, such as caloric restriction [17, 19], pharmacological activators (e.g., resveratrol or Thymoquinone) [16, 20], or genetic manipulation [21], may help reduce oxidative stress and preserve hearing function.

Cocoa beans from the *Theobroma cacao* tree are a significant source of polyphenols, particularly flavanols. Cocoa-based products are commonly consumed worldwide, making them natural dietary sources of antioxidants with therapeutic properties. Numerous studies have demonstrated that cocoa has beneficial effects against oxidative stress-related diseases by enhancing antioxidant enzyme activities and neutralizing free radicals [22, 23]. We have recently shown that a cocoa diet protects against ARHL in older C57Bl/6 J mice, significantly improving Auditory Steady-State Response (ASSR) [24].

In summary, SIRT1 and SIRT3 play crucial roles in mitigating oxidative stress, which is a key factor in the development of ARHL. Enhancing the activity of these Sirtuins through a cocoa-rich diet could offer a promising strategy to protect against ARHL. The current study intends to evaluate the intrinsic mechanisms involved in the protective effects of cocoa against age-induced oxidative damage in the inner ear and assess potential differences between sexes. To this end, the effect of the cocoa diet on SIRT-1, SIRT-3, p53, and FOXO3 expressions, oxidative stress generation, and oxidative damage to DNA was investigated. Furthermore, we studied the impact of cocoa on the activities of Superoxide Dismutase (SOD), Glutathione Peroxidase (GSH-Px), and Catalase (CAT) antioxidants enzymes. Finally, understanding the molecular basis of these sex differences in ARHL will accelerate the development of precision medicine therapies for ARHL.

## Materials and methods

### Experimental animals, diets, and design

One hundred adult C57BL/6 J mice, comprising both males and females at 3 months old, were procured from Charles River Laboratories (Wilmington, MA, USA). They were housed under standard conditions with a temperature range of 22–25 °C, 50% relative humidity, and 12-h light–dark cycles, with access to food and water ad libitum and daily monitoring. The mice were housed in groups of 4 per box.

Before the commencement of the experiment, mice were acclimatized for 7 days. Subsequently, they were randomly divided into two groups: the control group (*n* = 47) and the cocoa group (*n* = 47), where the latter’s diet was supplemented with cocoa at a dosage of 8.2 mg/kg body weight. The cocoa powder used, sourced from Chococru (London, UK), had a high polyphenol content. Cocoa doses were calibrated to match the daily intake equivalent to 5 g of cocoa for a 70 kg human according to an allometric scale [25]. Cocoa was incorporated into the standard feed at 8.2 g/kg, provided by Safe Diets (Augy, France), resulting in an approximate daily cocoa intake of 25 mg per mouse (considering their average consumption of about 3 g of pellets per day).

At 6 months of age, a subgroup of 24 mice (12 male, 12 female) underwent hearing assessment previously anesthetized with isoflurane (1.5–2%), constituting the 6-month study cohort. Once the tests were completed, these mice were sacrificed with inhalation anesthesia (5% isoflurane) followed by guillotine decapitation, and both cochleae were removed. This procedure was repeated for 14-month-old mice (24 mice, 12 males, 12 females) and 22-month-old mice (46 mice, 23 male, 23 female) equally divided between the control and cocoa groups cohorts. Six mice died during the study, resulting in a final count of 94. The mice belonged to the 22-month-old group, 4 to the standard diet group, and 2 to the cocoa group. The auditory study conducted with these mice has been previously published [24, 26].

### Extraction of protein homogenates of cochleae

Cochleae (*N* = 76) of mice of all experimental groups were used for different analyses such as ELISA, luminescent, and other assay kits. Thus, protein extracts of the whole cochlea were obtained by using T-PER™ (tissue protein extraction reagent) with the addition of a Complete mini protease inhibitor cocktail (ThermoFisher Scientific, Rockford, USA). The cochlea was disrupted for 2 min with a homogenizer ultra-Turrax T8 (IKA) followed by 1 min on ice. This treatment was repeated twice. The extract was centrifuged at 16.000 g for 10 min at 4 °C, and the supernatant was stored at − 80 °C. The protein concentration was determined by the BCA protein assay (ThermoFisher Scientific, Rockford, USA), following the manufacturer’s instructions.

### Histology and hematoxylin–eosin staining

Also, cochlea was used for histology and immunohistochemical study (*N* = 36). After fixation by intralabyrinthine perfusion of 4% paraformaldehyde (pH 7.4), cochleae were incubated in the same fixative overnight. Decalcification was performed in ethylenediamine tetraacetic acid solution (10% in phosphate-buffered saline) for 1–2 weeks. Subsequently, the tissues samples were dehydrated through a series of ethanol washes and incubated in Histo-Clear (Thermo Scientific, Rockford, USA). Following incubation in paraffin wax for 2 h at 60 °C, samples were embedded in wax blocks. The paraffin-embedded cochleae were cut into consecutive 3-μm-thick sections. They were rehydrated through Histo-Clear, graded ethanol, and H_2_O before staining with Hematoxylin and Eosin (H&E) to study histopathological damage to the cochlea. The samples were visualized and photographed using microscopy Olympus BX51 with different augmentations (Tokyo, Japan).

### Indirect immunofluorescence

Paraffin-embedded cochlea tissue sections were mounted on polysine-coated glass slides. Sections were irradiated with microwave in Trilogy™ (Cell Marque, Sigma-Aldrich, San Luis, USA) solution that deparaffinized, rehydrate, and unmask the tissue samples for 10 min and, afterwards, blocked at 37 °C for 2 h and incubated overnight at 4 °C with the anti-8OHdG antibody (Abcam, Cambridge, UK). Following washes, sections were incubated with the secondary Alexa Fluor 546-conjugated goat anti-rabbit antibody (1/250; Molecular Probes, Invitrogen, Thermo Scientific, MA, USA) for 45 min at 37 °C. Next, sections were incubated with 4′,6-diamidino-2-phenylindole (DAPI, 300 nM) at 37 °C for 5 min, reactive with fluorescent blue, marking the interlayer between DNA base pairs in the cell nuclei. Sections were then mounted and visualized under microscopy. The specificity of the immunostaining was evaluated by the omission of the primary antibody (negative controls). Olympus BX51 (Tokyo, Japan) was used to score tissue sections, and ImageJ, a free image-processing program, was used for quantitative image analysis. These experiments were performed in triplicate. The fluorescence intensity was quantified using the Image J program.

### Assay kits

Protein extracts from the cochleae were assayed by a different assay kit by the following proteins: Sirtuin 3 was quantified using an ELISA kit (SEE913Mu; Cloud-Clone Corp, Katy, USA); Sirtuin 1 levels were measured using an ELISA kit (E-EL-M0350; ELABSCIENCE, Houston, USA); Glutathione Peroxidase (GSH-Px) activity was measured using an assay kit (E-BC-K096-M; ELABSCIENCE, Houston, USA); Superoxide Dismutase (SOD) activity was measured using an assay kit (706002; Cayman Chemical, Michigan, USA); ROS assay kit (MBS2540517; MyBioSource, Inc., San Diego, USA); Catalase (CAT) assay Kit utilizes peroxidatic function of CAT for determination of enzyme activity (Cayman Chemical, Ann Arbor, MI, USA); FOXO3 used a Mouse FOXO3/FOXO3A ELISA Kit (LSBio company, Seattle, WA, USA) and Mouse p53 SimpleStep ELISA® Kit (Abcam, Cambridge, UK). The plates were evaluated in the FLUOstar Omega (BMG Labtech, Ortenberg, Germany) following the manufacturers’ instructions in each case. These experiments were performed in duplicate.

### ROS fluorometric assay kit

The total free radicals were measured by the ROS Fluorometric Assay Kit (MyBioSource, Inc., San Diego, CA, USA). The assay employs a specific fluorescent probe 2,7-dichlorofuorescin diacetate (DCFH-DA). In the presence of ROS, the DCFH-DA is oxidized to DCF (dichlorofluorescin) which is a strong green fluorescent substance. Fluorescence measurement was performed on a FLUOstar Omega (BMG Labtech, Ortenberg, Germany) plate reader (excitation to 502 nm and emission to 525 nm).

### ROS detection

Red fluorescent probe dihydroethidium (DHE; Calbiochem, San Diego, USA) was used to measure peroxynitrite anion (ONOO^−^), hydroxyl radical (^•^OH), or superoxide anion (O2^·−^) generation [27]. After dewaxing, sections of cochlea were incubated for 90 min at 37 °C with the DHE probe (4 μmol/l). In the presence of ROS, DHE is oxidized to ethidium and yields bright red fluorescence. Thereafter, samples were incubated with DAPI (300 nM) for 5 min at 37 °C. After, sections were mounted and visualized by fluorescence microscopy (Olympus BX51, Japan). The specificity of the staining was evaluated by the omission of the probe (negative controls).

### RNA/DNA extraction method

We used the UMSAgen method for simultaneous extraction of RNA and DNA from the cochlea due to the limited number of samples. The method we propose is based on the separation of RNA and DNA, taking advantage of the different solubility characteristics in the organic and aqueous phases. Once both the RNA and DNA were extracted from the samples, their purity was quantified and checked using NanoDrop™ One (Thermo Scientific, Waltham, MA, USA). The DNA/RNA was isolated from the cochleae of mice (*N* = 76, with 12 cochleae per group) of all age groups and diets.

### Measurement of DNA oxidation levels

The 8-hydroxy-20-deoxyguanosine (8-OHdG), a recognized marker of oxidative DNA damage, was measured using an Oxiselect™ Oxidative DNA Damage ELISA Kit (8-OHdG Quantitation) from Cell Biolabs (San Diego, USA). DNA was extracted from cochlear tissue samples using a UMSAgen method. The absorbance was read at 450 nm using a microplate reader FLUOstar Omega (BMG Labtech, Ortenberg, Germany). The concentrations of 8-OHdG were expressed in nanograms per milliliter.

### Quantitative real-time reverse transcription-polymerase chain reaction

The total RNA (1 μg) derived from the cochleae was reverse transcribed using a StaRT kit (AnyGenes, Paris, France) according to the manufacturer’s instructions, using Veriti Thermal Cycler (Applied Biosystems, Waltham, MA, USA).

A quantitative real-time reverse transcription-polymerase chain reaction was performed to study gene expression using the 7500 Fast real-time PCR detection system (Applied Biosystems,Waltham, MA, USA). Complementary DNA (cDNA) templates (2 μL) were added to 8 μL of Perfect Master Mix SYBRG (AnyGenes, Paris, France). The final volume was 10 μL. Polymerase chain reactions were carried out according to the procedures provided by the manufacturer in triplicate. The transcript levels were normalized to GAPDH and β-actin (used as reference genes). Determination of the relative expression levels was performed using the comparative “Ct” method. The following human primers were used in this study: Sirtuins 1 and 3, FOXO3, and p53 (AnyGenes, Paris, France).

### Statistical analysis

Statistical analysis was carried out using SPSS V. 19.0 software (IBM, Armonk, NY, USA). All data are expressed as mean ± deviation (SD). Comparisons between control and cocoa groups at each time point (6, 14, and 22 months) were performed using unpaired Student’s *t* tests. Sex-related differences within each group were also assessed using unpaired *t* tests. Statistical significance level was defined at *p* < 0.05.

### Ethical approval statement

All experimental procedures were approved by the Ethical Committee for Animal Experimentation of Comunidad de Madrid (PROEX 60.1/20). All institutional and national guidelines for the care and use of laboratory animals were followed. The authors have adhered to the ARRIVE guidelines. All experiments have been carried out by the 2010/63/EU Directive for animals.

## Results

### Cocoa attenuates cochlear structural changes in older mice

**Fig. 1:**
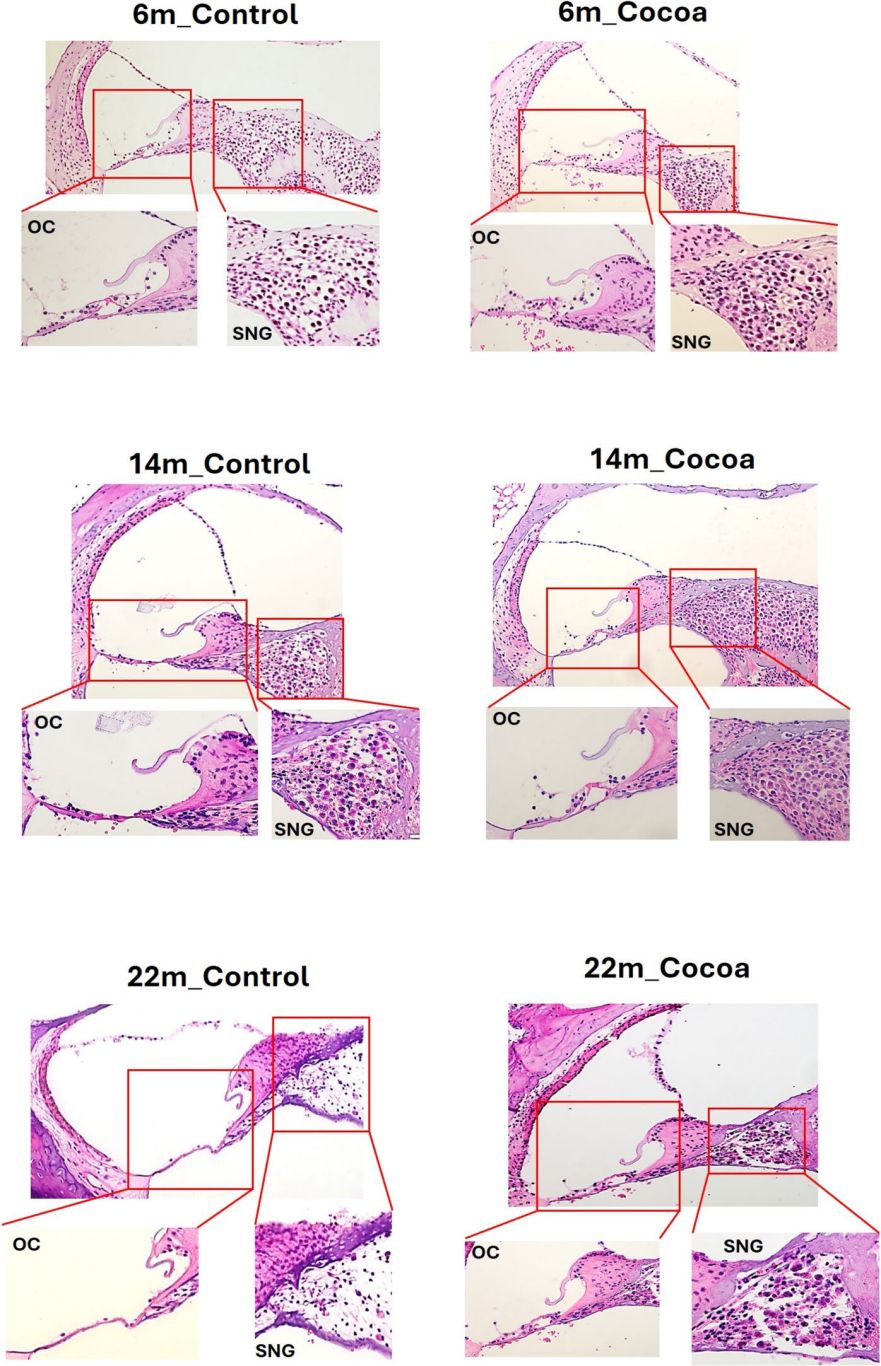
Representative images of degradation of hair cells of the organ of Corti (OC) and spiral ganglion nerve (SGN) cells in mouse cochleae detected through (H&E) staining. The OC and SGN cells are indicated. Scale bar 100 and 50 μm. (*n* = 6)

We performed histological analysis of cochlear cross-sections from all experimental groups of mice, and 6-month mice (Control and Cocoa groups) maintained a better overall cochlear cell structure than aged mice did. In contrast, in mice in control groups at 14 and 22 months, there was significant inflammatory cell infiltration, loss of inner hair cells, and severe spiral ganglion nerve (SGN) degeneration. These factors were significantly improved in the cocoa diets in the 14- and 22-month groups (Fig. 1). No morphological differences were observed in the cochlear structure between males and females.

### Cocoa diet increases SIRT1 and SIRT3 expression in the cochlea of older mice

**Fig. 2:**
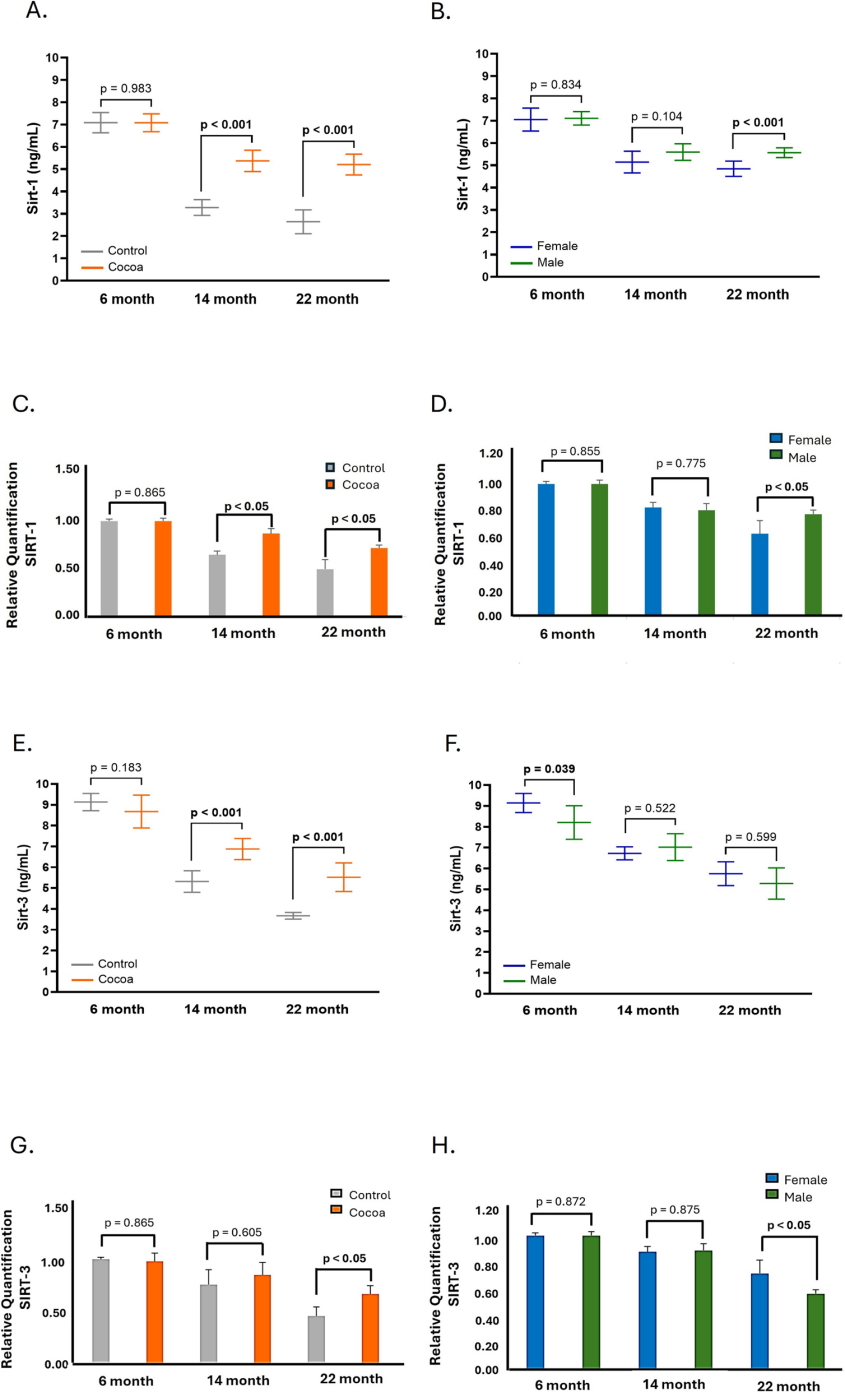
Cocoa increases the expression of SIRT-1 and SIRT-3 in the cochlea of mice. **A** SIRT-1 protein levels were determined by ELISA assay in cochlea lysates; **B** SIRT1 protein levels by sex in the cocoa groups; **C** gene expression of SIRT-1 in cochlea; **D** gene expression of SIRT-1 in cochlea by sex in the cocoa groups; **E** SIRT-3 protein levels were determined by ELISA assay in cochlea lysates; **F** SIRT-3 protein levels by sex in the cocoa groups; **G** gene expression of SIRT-3 in cochlea; **H** gene expression of SIRT-3 in cochlea by sex in the cocoa groups. Data are means ± SD of ng/mL (**A, B, E, F**), fold change (**C, D, G, H**) (*n* = 12). Reference genes: GAPDH and β-actin

Figure 2 shows that the protein levels (Fig. 2A) and gene expression (Fig. 2C) to SIRT-1 were significantly (*p* < 0.05) augmented in the cocoa group of mice 14 and 22 months of age compared with control groups.

The SIRT3 protein has behavior like SIRT-1. ELISA assay (Fig. 2E) and real-time PCR (Fig. 2G) showed identical data, with a significant decrease in SIRT-3 levels in control groups of old mice (*p* < 0.05), while levels increased from 14 months of age in mice with the cocoa diet, but only the cocoa group of 22-month mice showed a statistically significant increase in SIRT-3 expression (*p* < 0.05) (Fig. 2E, G).

Figure 2B, D, F, and H illustrates the differences in sex among animals within the cocoa group. Figure 2B indicates significant differences between males and females exclusively at 22 months of age (*p* < 0.001); specifically, SIRT-1 levels are markedly lower in females than males. However, no notable sex differences were detected concerning SIRT-3 levels. In control group animals, significant differences between males and females were identified at 22 months of age as detailed in Figure S1B of the supplementary material for SIRT-1 and SIRT-3. The expression patterns of the SIRT-1 and SIRT-3 genes align with the sex differences observed in protein levels (Fig. 2D, H; Figure S2B of supplementary material).

### Cocoa diet induces FOXO3 activation in the cochlea of older mice

**Fig. 3:**
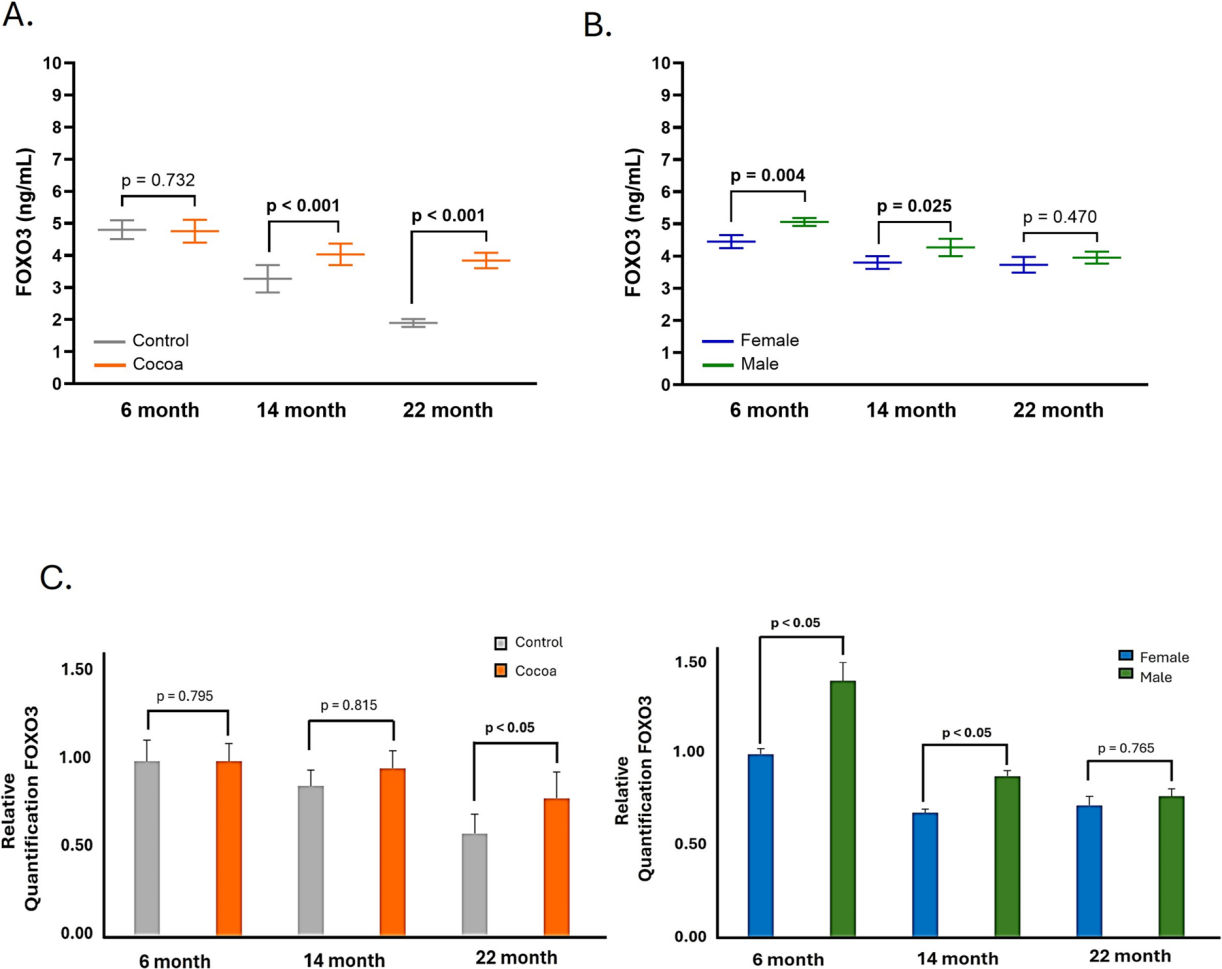
Cocoa increases expression of FOXO3 in the cochlea of mice. **A** FOXO3 protein levels were determined by ELISA assay in cochlea lysates; **B** FOXO3 protein levels by sex in the cocoa groups; **C** gene expression of FOXO3 in cochlea; **D** gene expression of FOXO3 in cochlea by sex in the cocoa groups. Data are means ± SD of ng/mL (**A, B**), and fold change (**C, D**). (*n* = 12). Reference genes: GAPDH and β-actin

To evaluate the effects of the cocoa diet on FOXO3 protein expression in cochlea, we measured its expression by real-time PCR and ELISA assay. Figure 3A, C showed that in the control groups of older mice, the expression of FOXO3 did not increase. On the contrary, in these same age groups, the cocoa diet increased the expression of FOXO3, being significant only in the 22-month-old group (Fig. 3A, C). If we consider sex as a variable, we found significant differences between males and females at 6 and 14 months of age in both the cocoa group (Fig. 3B, D) and the control group (Figure S1C, S2C of supplementary material).

### Cocoa diet modulates aging-increased p53 expression in cochlea

**Fig. 4:**
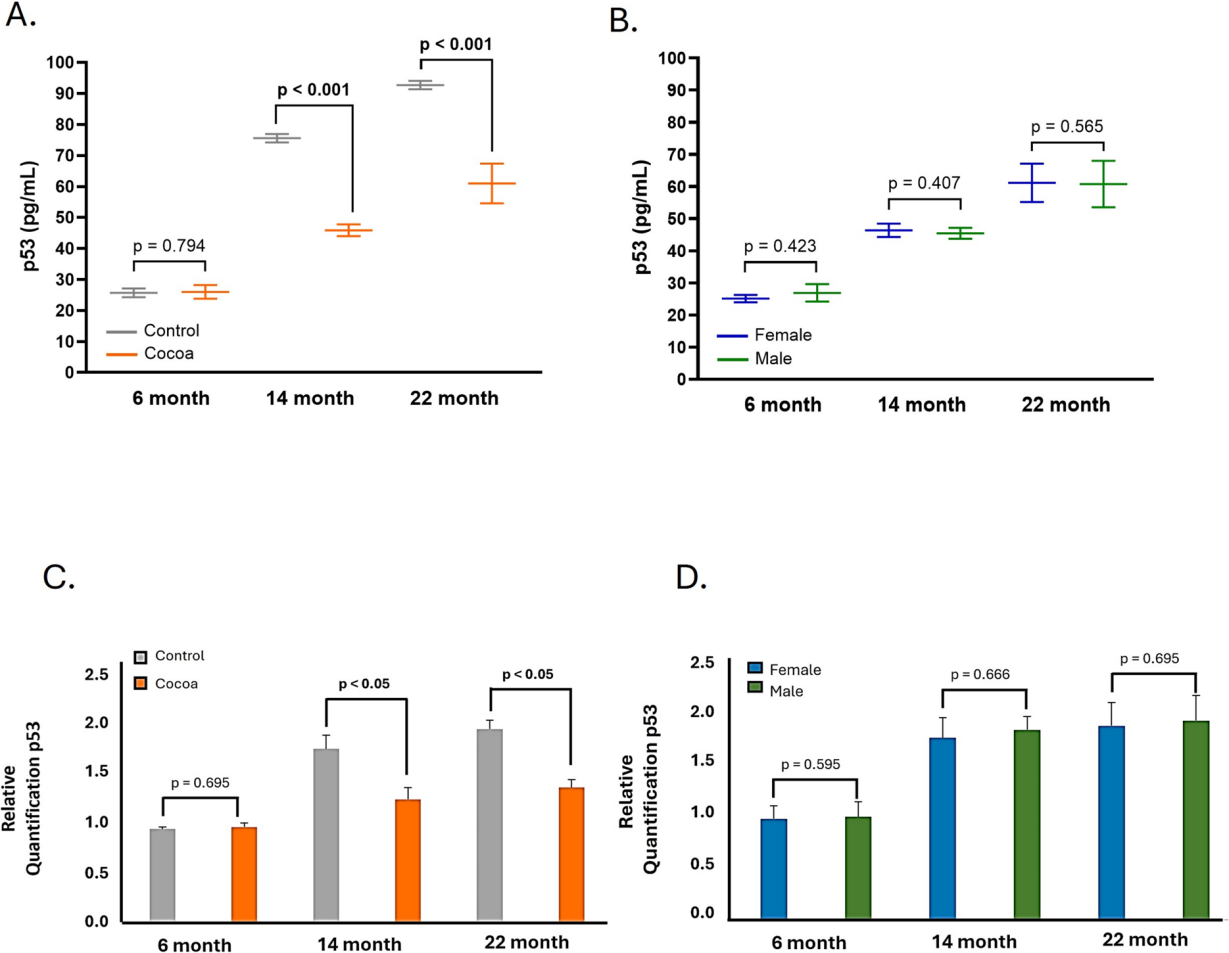
Cocoa increase expression of p53 in the cochlea of mice. **A** p53 protein levels were determined by ELISA assay in cochlea lysates; **B** p53 protein levels by sex in the cocoa groups; **C** Gene expression of p53 in cochlea; **D** gene expression of p53 in cochlea by sex in the cocoa groups. Data are means ± SD of ng/mL (**A, B**), and fold change (**C, D**). (*n* = 12). Reference genes: GAPDH and β-actin

Figure 4 shows the p53 expression in the cochlea, both at the protein (Fig. 4A) and gene levels (Fig. 4C). The cocoa diet shows a statistically significant decrease in p53 (*p* < 0.05) in 14- and 22-month-old mice compared to their control groups (Fig. 4A, C). No significant differences were identified concerning p53 between males and females in the group of mice with a rich-cocoa diet (Fig. 4B, D), and the same was observed in the group with a standard diet (Supplementary material: Figure S1D, S2D).

### Cocoa attenuated ROS generation in the cochlea of older mice

**Fig. 5:**
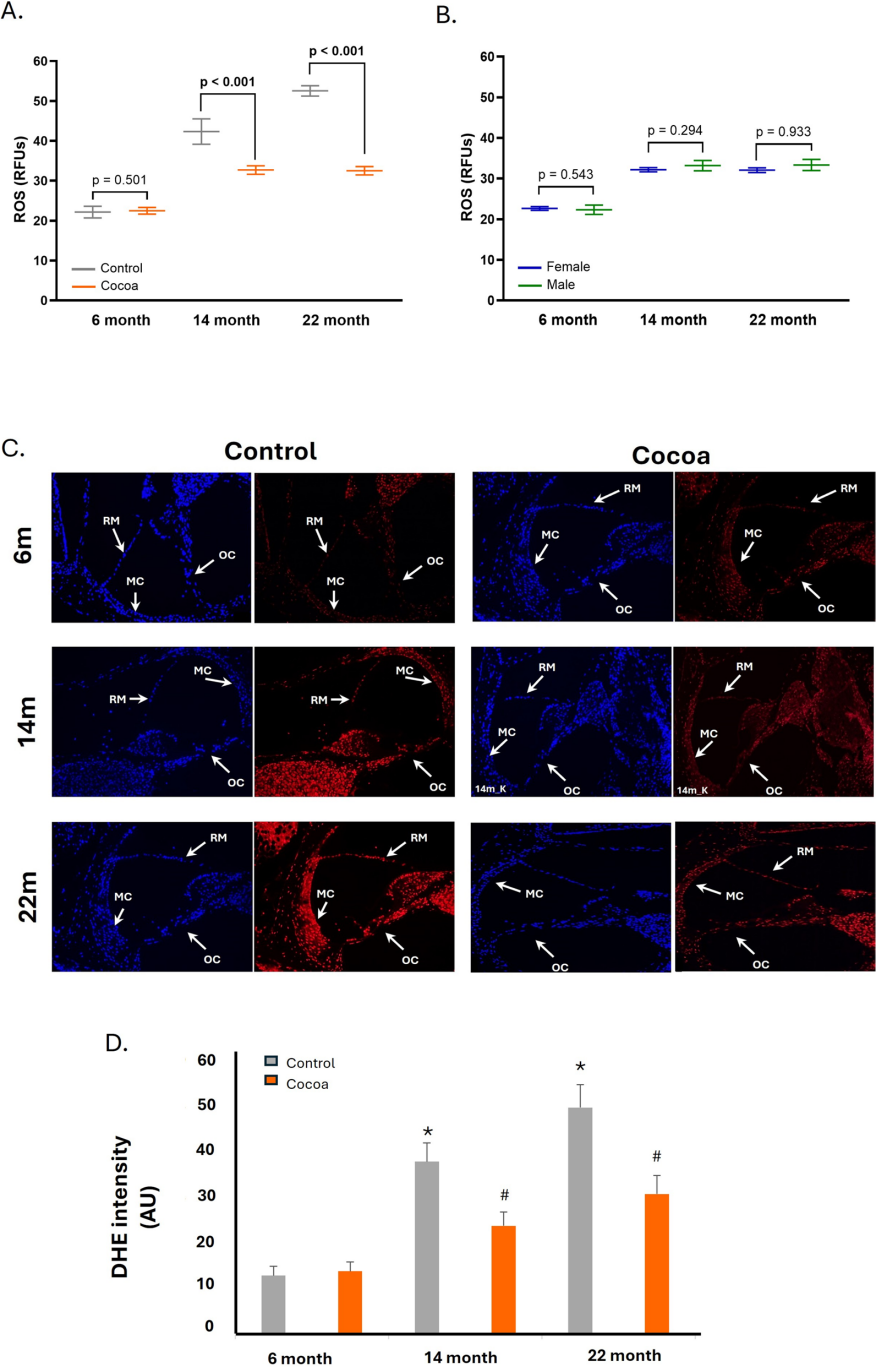
Cocoa modulated ROS production in cochlea mice. ROS were measured in cochlea tissue. **A** Comparison of ROS levels between the control group and the cocoa group across various ages; **B** comparison of ROS levels between female and male participants in the cocoa group; **C** detection of ROS with dihydroethidium fluorescent probe (red) in cochlea tissue of mice of different ages with and without cocoa-rich diet. Nuclei were detected in blue fluorescence by DAPI; **D** graphs represent the immunofluorescence intensity analyzed by Image J. Increased fluorescence is seen in several cell populations: marginal cells of Stria Vascularis (MC); Reissner’s membrane (RM) and Organ of Corti (OC). Scale bar 50 μm. The diagrams represent the mean ± SD of relative fluorescent units (RFUs) (**A, B**), and Arbitrary Units (AU) (**D**). (*n* = 12). *p < 0.05 vs. of 6 m-control group; ^#^*p* < 0.05 vs. respective control group

The next step was to evaluate the effect of the cocoa-rich diet on ROS production in cochlear tissue. As shown in Fig. 5A, ROS levels were high in the control groups of older mice (14 and 22 months), while low in the 6-month-old group. The cocoa-rich diet statistically significantly reduced this ROS generation in the 14- and 22-month-old groups. Figure 5B illustrates the absence of differences between sexes in ROS levels within the cocoa group. Conversely, in the standard diet groups, notable differences were observed between males and females at 22 months, with males exhibiting higher ROS levels (Figure S1E of the Supplementary Material).

The DHE probe detects ROS by visualizing a higher intensity of red fluorescence in cell nuclei. In the cochlear tissue of old mice (14 and 22 months) in the control group there is an increase in fluorescence in the cell nuclei compared to the control group of young mice (6 months) (Fig. 5C). The highest fluorescence intensity is observed in several cochlea structures, including the cells of the Organ of Corti, Reissner’s membrane, and marginal cells of the Stria Vascularis, indicating an increase in ROS. Due to cocoa intake, fluorescence intensity decreased in the old mice groups, reflecting a reduction in ROS production, but remained unchanged in the cocoa group of 6-month mice (Fig. 5C). Statistical significance was determined by analyzing fluorescence using ImageJ software (Fig. 5D). For the 14- and 22-month control groups, the DHE intensity increased, compared to the 6-month control group (*p* < 0.05). However, in these same age groups, but with the cocoa-rich diet, fluorescence intensity decreased significantly, i.e., ROS decreased (Fig. 5D).

### Cocoa diet prevents oxidative DNA damage in the cochlea of older mice

**Fig. 6:**
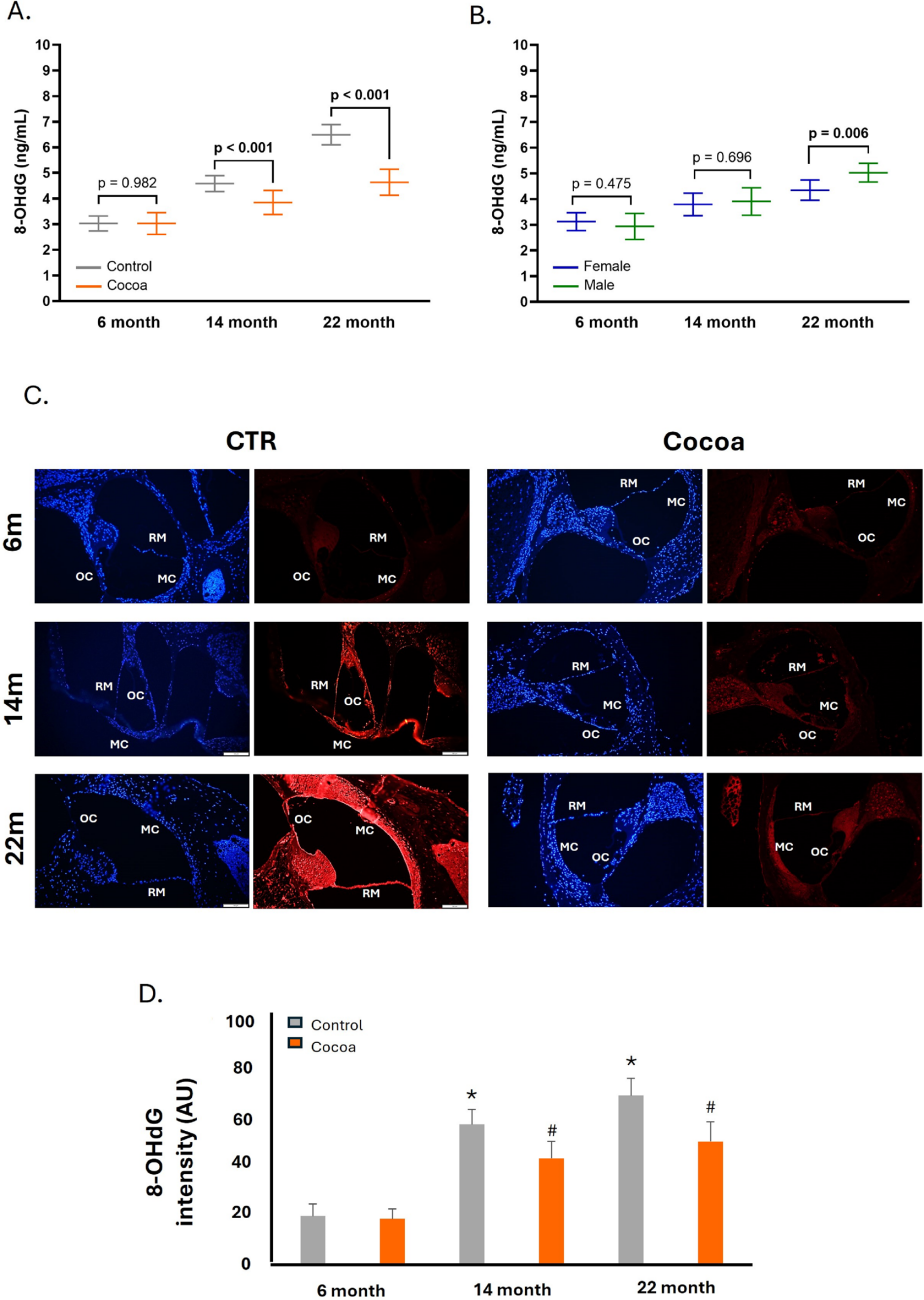
A rich cocoa diet prevents DNA oxidative damage in the cochlea of old mice. A colorimetric assay in cochlea protein extracts measured 8-OHdG levels, **A** comparison of 8-OHdG levels between the control group and the cocoa group across various ages and **B** comparison 8-OHdG levels between female and male participants in the cocoa group; The values represent the mean ± SD in ng/mL. (*n* = 12); **C** illustrative fluorescence images of immunostaining of 8-OHdG in cochlea tissue of mice of different ages with and without cocoa-rich diet. Nuclei were detected in blue fluorescence by DAPI, and 8-OHdG immunoreactivity was labeled in red fluorescence; **D** graphs represent the immunofluorescence intensity analyzed by Image J. Increased fluorescence is seen in several cell population: marginal cells of Stria Vascularis (MC); Reissner’s membrane (RM) and Organ of Corti (OC). Scale bar 50 μm. Data are means ± SD of Arbitrary Units (AU) (**D**). (*n* = 12). *p < 0.05 vs. of 6 m-control group; ^#^*p* < 0.05 vs. respective control group

An important increase in oxidative DNA damage happens during aging. The data showed a significant difference in the levels of 8-OHdG associated with the cocoa intake in the 14 and 22-month mice (Fig. 6A). This was due to a significant decrease in 8-OHdG levels with the cocoa diet in the 22-month group (e.g., 4.46 ± 0.21 ng/mL) when compared with the respective control group (e.g., 6.49 ± 0.2 ng/mL) (Fig. 6A). Figure 6B shows the statistically significant differences between males and females only in the 22-month cocoa group. No variations were observed in the standard diet group (Figure S1F, supplementary material).

The data obtained are consistent with the immunofluorescence identification of the 8OH-oxodG protein (red), which exhibited a statistically significant increase in the control groups at 14 and 22 months (Fig. 6C, D) when compared to the control group at 6 months (*p* < 0.05). Furthermore, the fluorescence intensity diminished in the groups that received dietary supplementation with cocoa. No differences were observed between the 6-month group with cocoa and the 6-month group without cocoa (Fig. 6C, D).

### Cocoa diet modulated antioxidant enzyme activities in the cochlea of older mice

**Fig. 7:**
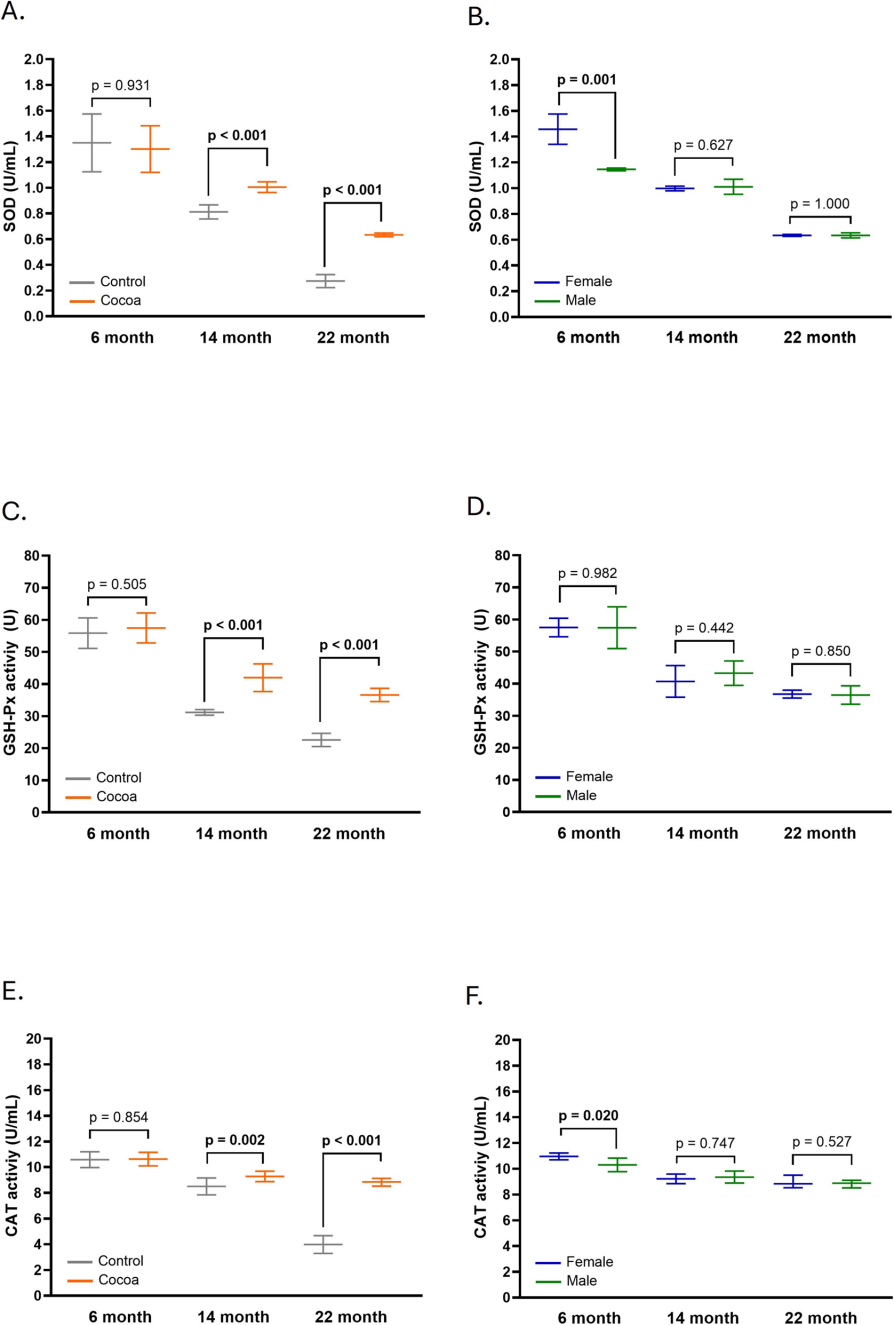
Cocoa modulated the total antioxidant response in cochlea aged mice. **A** Comparison of SOD levels between the control group and the cocoa group across various ages; **B** comparison SOD levels between female and male participants in the cocoa group; **C** comparison of GSH-Px activity between the control group and the cocoa group across various ages; **D** comparison GSH-Px activity between female and male participants in the cocoa group; **E** comparison of CAT levels between the control group and the cocoa group across various ages; **F** comparison CAT levels between female and male participants in the cocoa group. The enzymes were measured in whole mice cochlear extracts from animals at different ages with and without a cocoa-rich diet. (*n* = 12)

To determine the effect of the cocoa diet on three antioxidant systems in the cochlear tissue of mice, we determined SOD, GSH-Px, and CAT activities. Cocoa intake increased SOD activity (*p* < 0.05) in the 14-month group, going from 0.63 ± 0.05 to 0.81 ± 0.08 (U/mL) (Fig. 7A). In the case of CAT and GPx, the diet with cocoa also significantly increased their activities, for example, for the 22-month-old group, CAT activity went from 6.59 ± 0.3 to 9.06 ± 0.4 (U/mL) (Fig. 7C) and GPx activity went from 22.6 ± 0.5 to 36.7 ± 0.8 (U) (Fig. 7E).

Furthermore, we demonstrated that within the 6-month-old cocoa group, there were significant differences between males and females in the activity of the SOD (Fig. 7B) and CAT (Fig. 7F) enzymes. However, no significant sex-related differences in GPx activity were observed at any age group (Fig. 7D). This trend is also evident in the control groups (Figure S3A, S3C of the supplementary material).

## Discussion

Numerous pieces of evidence indicate that robust antioxidant activity is crucial in delaying ARHL [27, 28]. Current research on preventing and treating ARHL is heavily focused on reducing oxidative damage and restoring the balance in the oxidative/antioxidant system [27, 28]. Therefore, cocoa, with a strong antioxidant effect, could be a good option as a treatment for ARHL. Our group has recently shown, for the first time, the otoprotective effect of cocoa against ARHL. These findings show significantly improved ASSR auditory thresholds in mice on a cocoa diet across the frequencies measured (from 4 to 32 kHz), suggesting a potential link between the enhanced hearing data and the presumed protection of the cochlear tissue [24]. This study was carried out in the same mice as the present investigation, in which the cochlea was removed after hearing tests. However, the molecular mechanisms by which cocoa prevents ARHL remain unknown; therefore, this was our next objective. This study demonstrated that a cocoa-rich diet increases the expression of SIRT1, SIRT3, and FOXO3, which play a critical role in inhibiting p53 in the cochlea of ​​aged mice. Furthermore, we demonstrate that cocoa protects against cellular damage of the cochlea due to aging, by modulating the production of ROS, decreasing oxidative DNA damage, and increasing the level of antioxidant enzymes, such as CAT, GPx, and SOD. Finally, for the first time, the effect of sex has been determined for the different biomarkers analyzed in mice cochlea, demonstrating that there are differences between males and females in some of them.

The results we obtained on SIRT1 and SIRT3, which mediate the otoprotective effects of the cocoa diet against oxidative damage to the cochlea, are consistent with the anti-stress and anti-aging effects of Sirtuins [29, 30]. SIRT1 is crucial in protecting against DNA damage and cellular oxidative stress [31]. Our data demonstrated that the cocoa diet increased SIRT1 expression by reducing oxidative DNA damage and ROS generation in the cochlea of ​​old mice, suggesting a decrease in hearing loss. Related to our data, Pang et al. [16] compared 2- and 12-month-old C57BL/6 mice, showing that SIRT1 expression was greatly decreased in older mice. However, diet supplementation with resveratrol compared with the normal-fed mice of the same age increased the SIRT1 level in the cells of the cochlea of mice and improved the ARHL [16]. Recent studies have shown that increased SIRT1 overexpression in mice by the transgenic technique confirmed that this could protect cochlear hair cells and improve ARHL [15]. In 2021, Song et al. [18] exposed Sprague–Dawley rats to environmental enrichment (EE) for 12 weeks. They showed that EE increased SIRT1 activity in the auditory cortex and improved ARHL [18]. In another study, a high-fat diet reduced SIRT1 expression in the cochlea and got worse ARHL, while the treatment with N1-methylnicotinamide increased SIRT1 and improved ARHL [19]. In contrast, Han et al., [32] indicated that SIRT1 deficit activated FOXO3a, improved the survival of cochlear hair cells to ROS damage, and delayed the development of ARHL [32].

Recent research indicated that SIRT3 is vital to maintaining mitochondrial function and protecting against oxidative stress. It achieves this primarily by regulating antioxidant enzymes crucial for neutralizing ROS and preventing cellular damage [33, 34]. Consistent with these studies, our results demonstrated, for the first time in the cochlea of old mice, that a cocoa-rich diet, through activation of the SIRT3 signaling pathway, regulated ROS homeostasis, mediated by reducing DNA oxidative damage, decreasing ROS generation, activation of FOXO3A, and increased antioxidants enzymes, such as SOD, CAT, and GPx. In line with our findings, Zeng et al. [35] demonstrated using a D-galactose-induced aging rat model that decreased SIRT3 levels reduce superoxide dismutase 2 (SOD2) activity. This reduction leads to increased ROS generation, which accumulates with aging, causing mitochondrial dysfunction and increased apoptosis of auditory cells, ultimately resulting in ARHL. Someya et al. [17] demonstrated that the Sirt3-mediated modulation of the glutathione antioxidant defense system plays a central role in the reduction of ROS in cochlear tissue under Caloric Restriction (CR) conditions. Also, it showed that CR reduced oxidative DNA damage in the cochlea from Wild Type (WT) mice but did not in Sirt3^−/−^ mice. CR also increased hair cell and spiral ganglion neuron survival in the WT, but not in Sirt3^−/−^ mice. In human umbilical vein endothelial cells (HUVECs), Zhou et al. [36] found that pretreatment with Resveratrol suppressed tert-butyl hydroperoxide (t-BHP)-induced oxidative damage by an increase of the enzymatic activities of SOD2, isocitrate dehydrogenase 2 (IDH2), and GSH-Px. Moreover, Resveratrol significantly reduced mtROS production by stimulating Sirt3 expression within the mitochondria and subsequent upregulation of FOXO3A.

Given that the p53 protein is a crucial regulator of various pathways involved in [37], we studied whether the cocoa diet affects p53 expression in the cochlear tissue of old mice. Our research revealed that the p53 expression was higher in the cochlea of old mice (14 and 22 months of age) compared to young mice (6 months of age) under a standard diet, and this increase was significantly reduced with the preventive intake of cocoa. These findings align with prior studies showing that Luteolin regulates p53 phosphorylation through SIRT1 in auditory cells HEI-OC1 senescent cells [38], and that Salidroside exerts a protective effect by regulating redox and p53/p21 expression in senescence [39]. Dong et al. [40] investigated the effects of Erlong Zuoci decoction (ELZCD, a typical traditional Chinese medicine based on plants) on the ARHL in C57BL/6 J mice. The RT-PCR results showed that the p53 expression in the ARHL group increased compared to that of the control group, and ELZCD reduced the elevated p53 expression which improved hearing. Also, the study of Kim et al. [41] demonstrated that SIRT1 and SIRT3 activation promoted inhibition of NF‐κB, IDH2, and p53, leading to a maintained reduction of apoptosis and inflammation, supported normal mitochondrial function, and finally protective effects against ARHL in C57BL/6 mice treated with β‐Lap, a quinone‐containing natural compound (3,4‐dihydro‐2,2‐dimethyl‐2H‐naphtho [1,2‐b] pyran‐5–6‐dione).

The interplay between SIRT-1, SIRT-3, FOXO3, p53, and ARHL is a dynamic process involving the regulation of oxidative stress responses, mitochondrial function, and apoptosis. By modulating the activity of p53 and enhancing cellular resilience to oxidative stress, SIRT-1 and SIRT-3 offer protective effects against ARHL. Understanding these interactions can provide insights into potential therapeutic targets for mitigating ARHL and promoting healthy aging.

Historically, basic and preclinical research has largely overlooked the influence of sex as a biological variable (SABV). In many studies, the sex of the animals or participants was either unspecified or research was conducted exclusively on one sex—typically male—while if the findings would apply to both sexes. It has long been recognized that ARHL is more prevalent [42, 43] and more severe [44, 45] in men than in women. While auditory threshold deterioration in men can begin as early as the second or third decade of life, women often experience symptoms much later—several years [45] or even decades afterwards [46]. Understanding the role of SABV in ARHL is essential for uncovering the underlying pathophysiology and enhancing therapeutic strategies. Therefore, in this study, we determined the differences between sexes for each of the biomarkers analyzed.

The observed sex-related differences in antioxidant enzyme activity (e.g., SOD and CAT) and in molecular markers such as SIRT1, SIRT3, FOXO3, 8-OHdG, and ROS may be influenced by sex hormones, particularly estrogens and androgens. Estrogens are well known to exert protective effects against oxidative stress by regulating the expression and activity of antioxidant enzymes, reducing mitochondrial ROS production, and enhancing mitochondrial biogenesis [47, 48]. For instance, 17β-estradiol increases the expression of SOD, CAT, and GPx via estrogen receptor-mediated pathways, contributing to better redox homeostasis in females [49]. These mechanisms may account for the higher levels of some antioxidant enzymes observed in females compared to males in our study.

In addition to classical antioxidants, sex hormones may modulate the activity and expression of SIRT1 and SIRT3, which play key roles in cellular stress responses, mitochondrial regulation, and aging. Estrogen has been shown to activate SIRT1, thereby influencing FOXO3-mediated antioxidant gene expression and promoting cell survival pathways [50]. Similarly, SIRT3, a mitochondrial deacetylase, has been reported to respond to estrogen signaling to regulate mitochondrial ROS detoxification and energy homeostasis [51]. This suggests that estrogen may enhance SIRT3-dependent mitochondrial antioxidant defense, contributing to sex differences in aging-related hearing loss.

Moreover, FOXO3, a downstream target of both SIRT1 and SIRT3, is involved in the transcription of antioxidant genes and the suppression of apoptosis. Estrogen has been shown to upregulate FOXO3 activity in various tissues, thereby promoting longevity-associated gene expression [52]. This pathway may underlie some of the observed sex differences in cochlear protection.

In contrast, the tumor suppressor protein p53, modulated by cocoa in our study, could also be influenced by sex hormones, although no differences between sexes were found in our study. Several studies have shown that estrogen inhibits p53-mediated apoptosis and oxidative damage, possibly through SIRT1 activation [53]. Conversely, testosterone can promote increased p53 activity and proapoptotic signaling under certain stress conditions [54], which could partly explain the greater vulnerability of males to age-related cochlear damage.

These hormonal influences collectively suggest that biological sex modulates the response to oxidative stress and aging at multiple molecular levels. Therefore, sex-specific regulation of SIRT1, SIRT3, FOXO3, 8-OHdG, ROS, and antioxidant enzymes may contribute to the differential susceptibility to age-related hearing loss observed between males and females. These findings underscore the importance of considering sex as a biological variable when evaluating the efficacy of antioxidant-based interventions, such as cocoa supplementation, in age-related diseases.

In conclusion, this study provides data demonstrating that a cocoa-rich diet has protective effects against aging damage in mice. The upregulation of SIRT1, SIRT3, and FOXO3 expression by cocoa plays a pivotal role in inhibiting p53 phosphorylation and reducing oxidative stress in the cochlea. Furthermore, for the first time, sex differences have been identified in some of these biomarkers, so understanding the molecular basis of sex differences in ARHL will accelerate the development of precision medicine therapies for ARHL. These results indicate that cocoa could be a promising natural compound for mitigating health issues associated with age-related diseases, such as ARHL.

## Limitations

Despite the promising findings of this study, several limitations should be acknowledged. First, the investigation employed a single cocoa dosage (8.2 mg/kg body weight), which was calibrated to match a human-equivalent dose of 5 g of cocoa for a 70 kg adult using an allometric scaling approach. While this enhances translational relevance, it does not consider potential dose–response relationships that could clarify the optimal therapeutic range.

Secondly, cocoa’s polyphenol content is highly variable and depends on several factors such as cultivar, geographical origin, fermentation, and processing methods. This heterogeneity could impact the reproducibility of results and the standardization of bioactive compound intake across studies. In addition, polyphenols exhibit diverse bioavailability and absorption profiles. Compounds such as epicatechin and catechin—major flavanols in cocoa—are rapidly absorbed in the small intestine but undergo extensive metabolism in the liver and gut microbiota, affecting their systemic bioactivity. The pharmacokinetics, including peak plasma concentrations and half-lives, vary widely between polyphenol subtypes, potentially influencing their tissue-specific effects, including in the cochlea. These metabolic transformations can modulate both their antioxidant capacity and their interaction with molecular targets such as SIRT1, SIRT3, FOXO3, etc. Therefore, understanding the bioefficacy of cocoa in vivo requires a deeper analysis of the absorption, distribution, metabolism, and excretion (ADME) of its polyphenols.

Lastly, although the mouse model provides valuable insights into the mechanisms underlying ARHL, translating these findings to humans remains challenging. Differences in auditory system anatomy, metabolism, and lifespan between species may limit the direct applicability of the results. Clinical trials are therefore essential to confirm whether the observed molecular and functional benefits of cocoa extend to human aging and hearing.

## Supplementary Information

Below is the link to the electronic supplementary material.

Supplementary file1 (TIFF 1451 KB)

Supplementary file2 (TIFF 2277 KB)

Supplementary file3 (TIFF 823 KB)

## Data Availability

Data available on request due to restrictions on privacy. The data presented in this study are available on request from the corresponding author.
