# Supplementary figures and images for: Sirtuins mediate the reduction of age-related oxidative damage in the cochlea under a cocoa-rich diet
Source: GeroScience. 2025 Aug 20;48(2):2987–3006. doi: 10.1007/s11357-025-01847-8 (PMC12972465; doi:10.1007/s11357-025-01847-8)

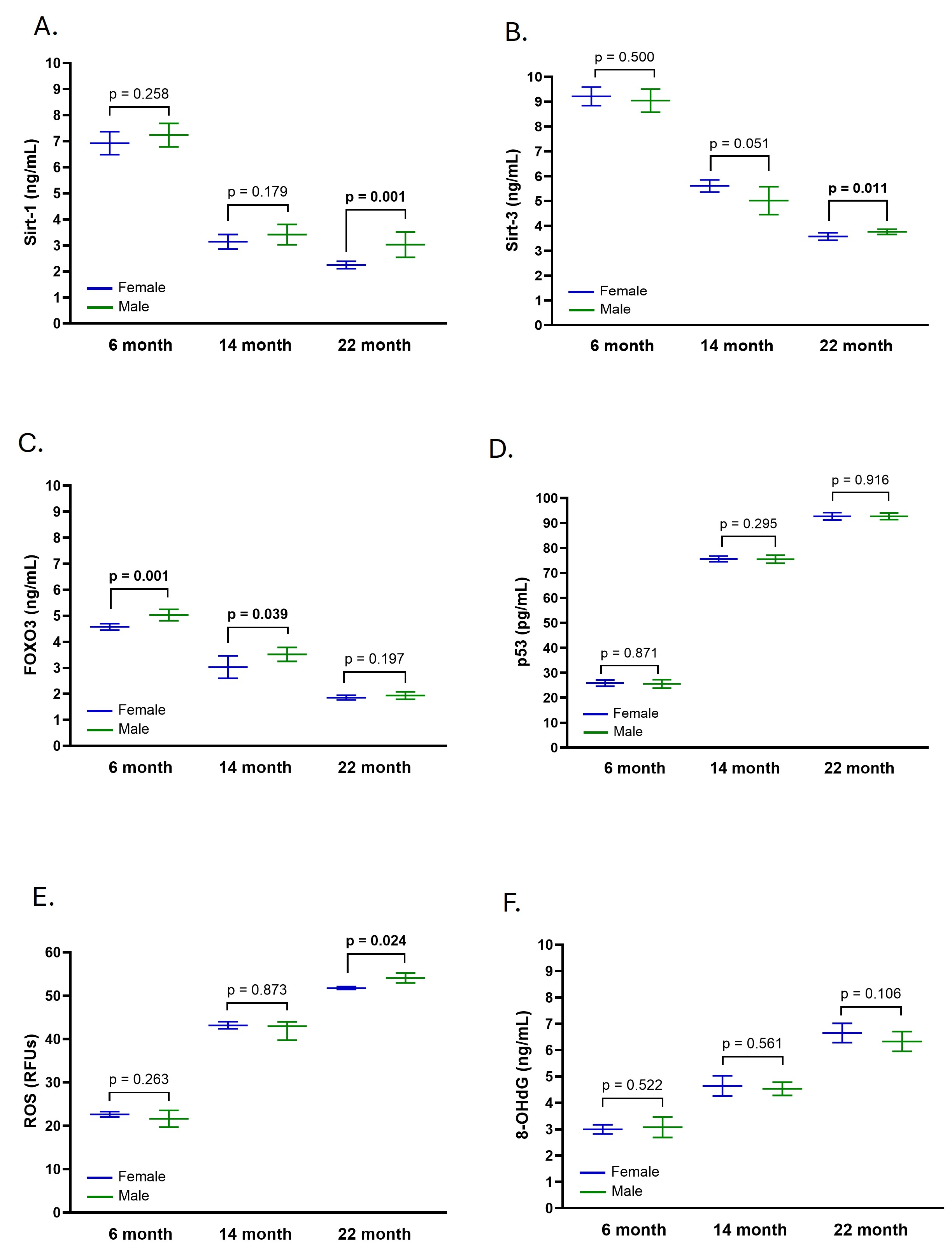

Supplement: Supplementary file 1 — Supplementary file1 (TIFF 1451 KB) [file 11357_2025_1847_MOESM1_ESM.tiff]

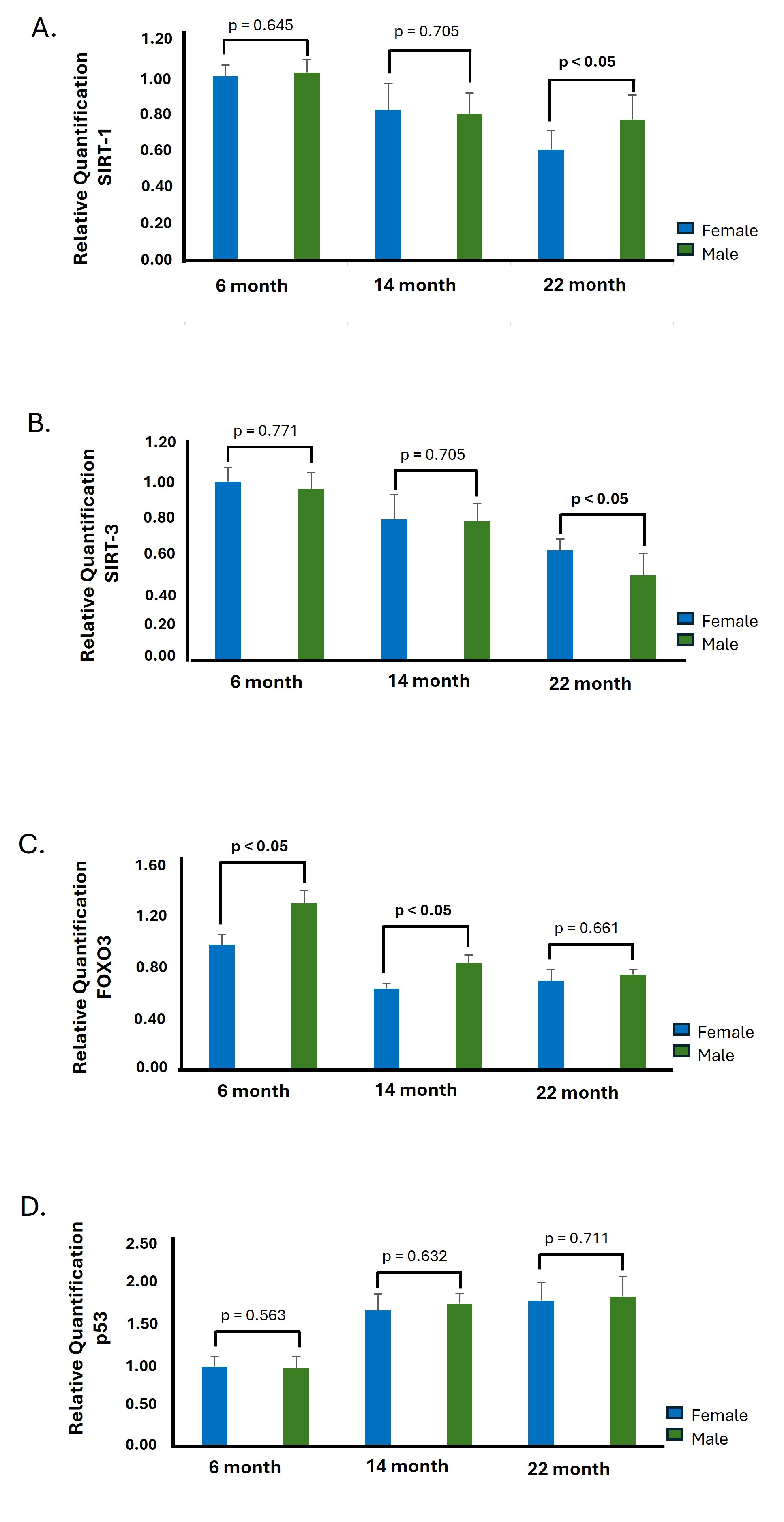

Supplement: Supplementary file 2 — Supplementary file2 (TIFF 2277 KB) [file 11357_2025_1847_MOESM2_ESM.tiff]

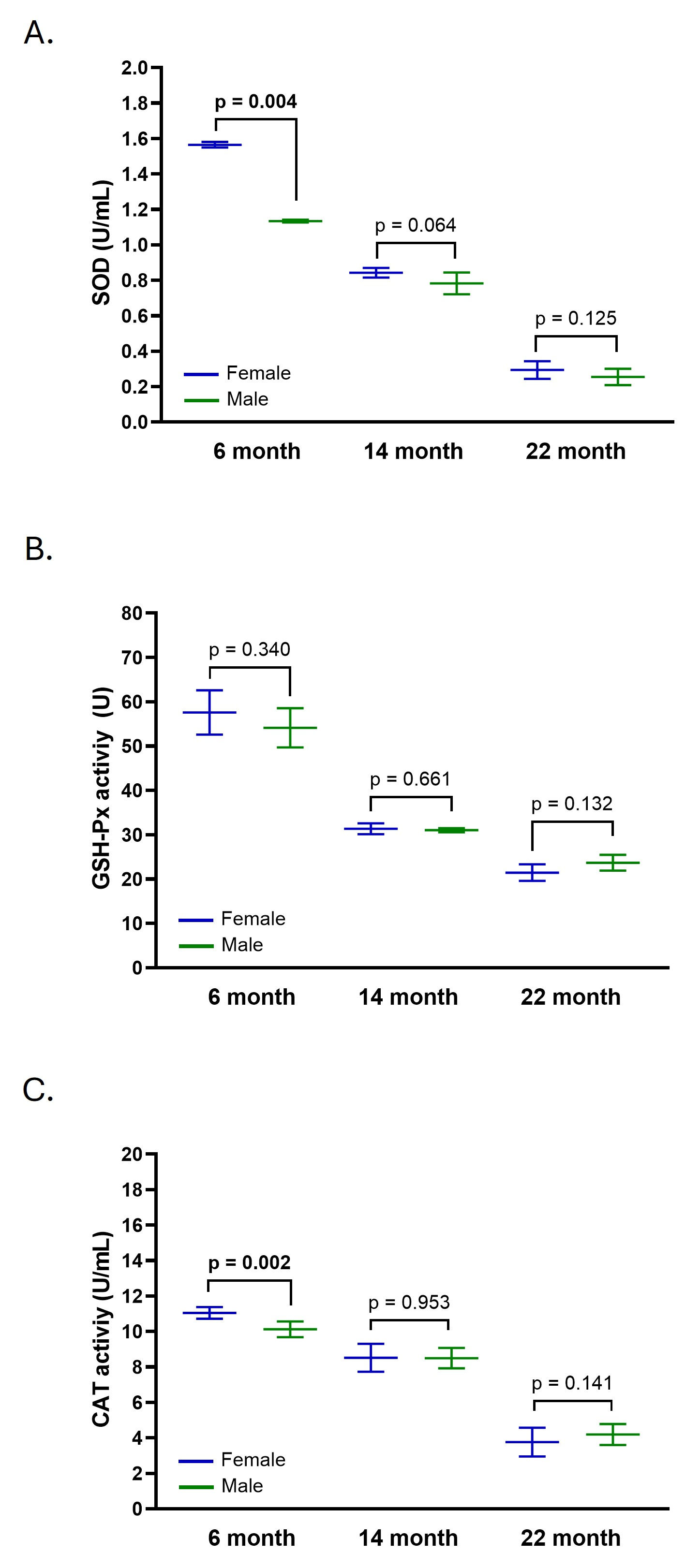

Supplement: Supplementary file 3 — Supplementary file3 (TIFF 823 KB) [file 11357_2025_1847_MOESM3_ESM.tiff]
